# A 65-Year-Old Male With Classic Cardiac Sarcoidosis: Case Report and Review of the Literature

**DOI:** 10.7759/cureus.31705

**Published:** 2022-11-20

**Authors:** Natalia Moguillansky, Ronny Samra, Peter A Drew, Diego Moguillansky

**Affiliations:** 1 Department of Medicine, Division of Pulmonary Critical Care and Sleep Medicine, University of Florida Health, Gainesville, USA; 2 College of Medicine, University of Florida, Gainesville, USA; 3 Department of Pathology, University of Florida, Gainesville, USA; 4 Department of Pediatrics, Cardiovascular Medicine, and Internal Medicine, University of Florida Health Congenital Heart Center, Gainesville, USA

**Keywords:** cardiac fdg-pet, mri cardiac, cardiac mri, transthoracic echocardiogram, cardiac sarcoidosis

## Abstract

Sarcoidosis is a systemic disease characterized by the formation of non-necrotizing granulomas, primarily involving the lungs and other organs such as the heart. The diagnosis of cardiac sarcoidosis can be difficult. The last set of diagnostic guidelines for diagnosis and treatment of cardiac sarcoidosis was published in 2019 by the Japanese Circulation Society (JCS).

We describe a case of classic cardiac sarcoidosis and review the literature on clinical presentation, imaging, and management.

## Introduction

Sarcoidosis is a systemic disease characterized by the formation of non-necrotizing granulomas, primarily involving the lungs (the prevalence of pulmonary involvement in patients with sarcoidosis is 90%) and other organs such as the heart (prevalence is 5-10%) [1, 2]. Initial screening for cardiac sarcoidosis includes clinical evaluation and ECG. If clinical suspicion is high or the ECG is abnormal showing repolarization abnormalities, arrhythmias or conduction disease, then echocardiography, cardiac MRI, and if still unclear, cardiac PET are recommended. Management includes treatment of the arrhythmias, heart failure and immunosuppression, as well as consideration for permanent pacing or placement of an automatic implantable cardioverter defibrillator (AICD). This is a case report and literature review by PubMed search, as such focuses on highlighting the typical presentation of cardiac sarcoid as well as the clinical utility of various diagnostic studies. The conclusions are consistent with our literature review.

## Case presentation

A 65-year-old man with a history of obesity, hypertension, and type 2 diabetes mellitus was referred to our institution for the management of non-tuberculous mycobacterial pulmonary infection in March 2020. Two years prior to his presentation he developed dyspnea on exertion and a dry cough.

He was evaluated at an outside institution with a PET CT scan which showed abnormal lymphadenopathy in the neck, thorax, and abdomen with increased Standardized Uptake Value (SUV). A prior biopsy of a cervical lymph node at that time was negative for malignancy and showed granulomatous inflammation. Concurrently, his sputum culture grew Mycobacterium avium complex (MAC) and he was started on azithromycin and clofazimine for suspected disseminated MAC. His family history was significant for heart disease in his father. He denied any history of tobacco or alcohol use.

On presentation his oxygen saturation was 95% on room air, his blood pressure was 197/89 mmHg with a pulse of 77 beats per minute, and body mass index was 44. On general examination, he was in no acute respiratory distress and was fully alert and oriented. His lungs were clear to auscultation bilaterally and general examination of both the cardiac and nervous systems was unremarkable.

**Figure 1:**
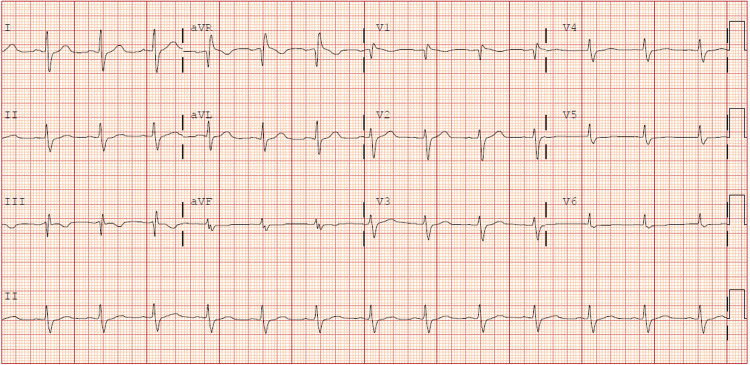
ECG showing sinus rhythm with right bundle branch block and secondary repolarization changes.

ECG on presentation showed sinus rhythm with right bundle branch block and secondary repolarization changes (Figure 1).

**Figure 2:**
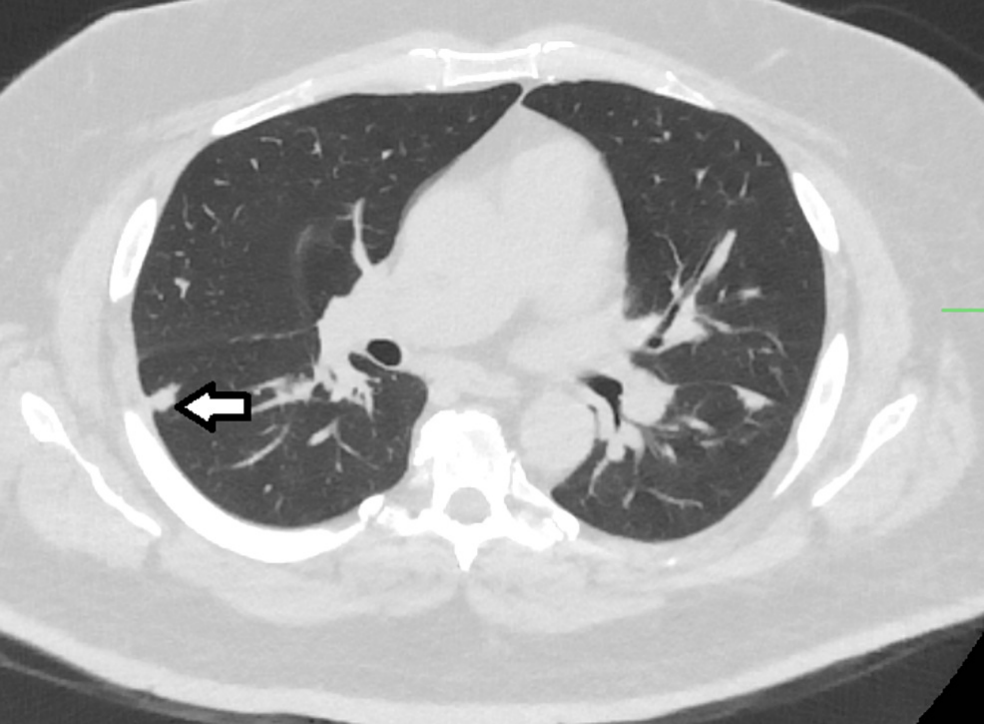
CT chest (lung window) (axial view). Multiple subcentimeter pulmonary nodules, Right lower lobe nodule (arrow).

**Figure 3:**
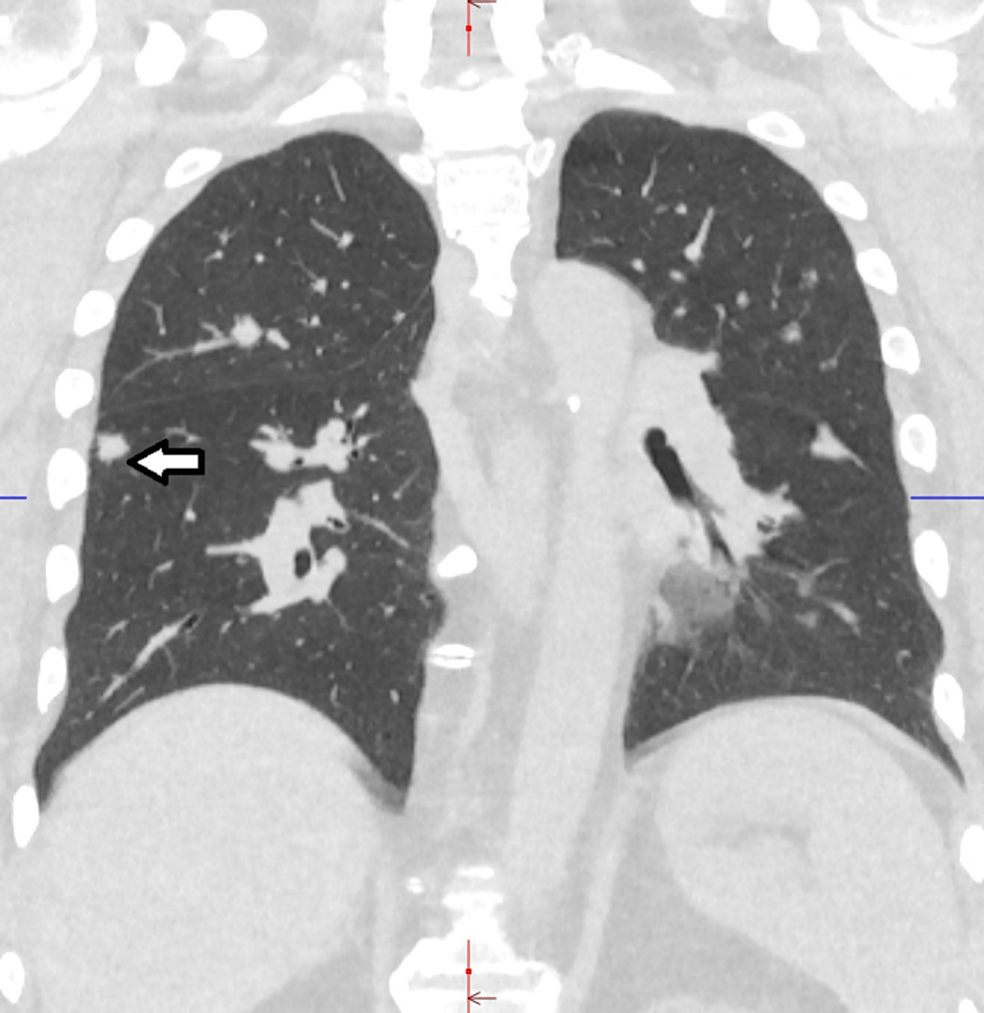
CT chest (lung window) (coronal view). Right lower lobe nodule (arrow).

**Figure 4:**
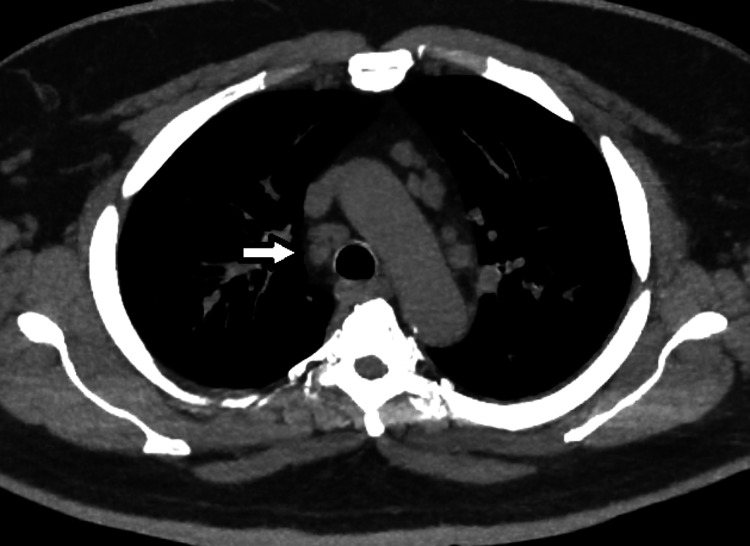
CT chest (mediastinal window) above the carina. Mediastinal lymphadenopathy (arrow).

CT scan of the chest showed multiple, bilateral, sub-centimeter lung nodules (Figures 2, 3) and mediastinal lymphadenopathy (Figure 4). There were no infiltrates, cavitary lesions, or evidence of interstitial lung disease.

Bronchoalveolar lavage was also performed and cultures were negative for bacterial, fungal, and acid-fast organisms.

Whole body PET CT scan showed innumerable enlarged head, neck, mediastinal peri-bronchial, and paratracheal nodes, with additional involvement of retroperitoneal, periaortic, and inguinal nodes. SUV uptake was between 3.5 to 12.5. Additionally, diffuse multifocal pulmonary infiltrates with 18F-fluorodeoxyglucose (FDG) uptake were present throughout the bilateral lung fields, as well as slightly increased multifocal nodular uptake throughout the liver and spleen. Interestingly, there was additional nodular uptake within the myocardium, suggestive of cardiac involvement.

**Figure 5:**
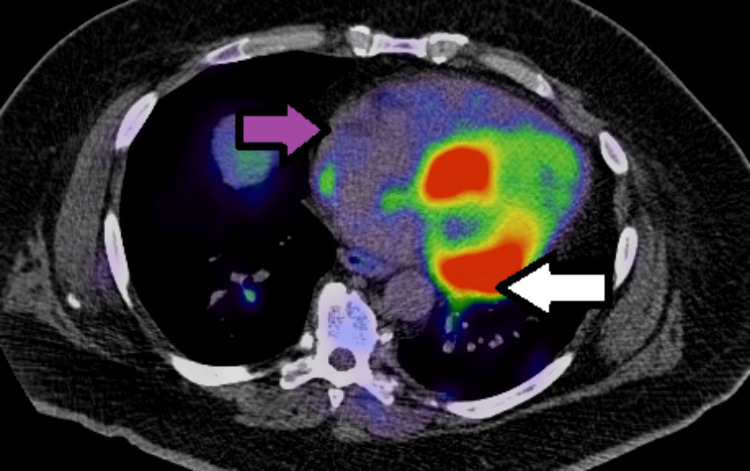
Cardiac PET CT Nodular SUV uptake within the myocardium (white arrow); No SUV uptake (purple arrow). SUV: Standardized uptake value

A cardiac PET CT scan demonstrated patchy uptake of FDG (Figure 5) in the myocardium primarily in the basal anterior wall and the basal inferolateral wall, matching areas of significant fibrosis seen on cardiac MRI. Right ventricular (RV) uptake was seen as well.

An echocardiogram showed mild dilatation of the left ventricle (LV) with moderate left ventricular systolic dysfunction and global hypokinesis [ejection fraction (EF) 30-40%] (Video 1). Grade 2 diastolic dysfunction was also noted, but an adequate estimation of the right ventricular pressure could not be obtained.

**Video 1:** Four chamber echocardiographic cine Left ventricular dilation with moderate left ventricular dysfunction. Right ventricular size and function appear normal.

**Figure 6:**
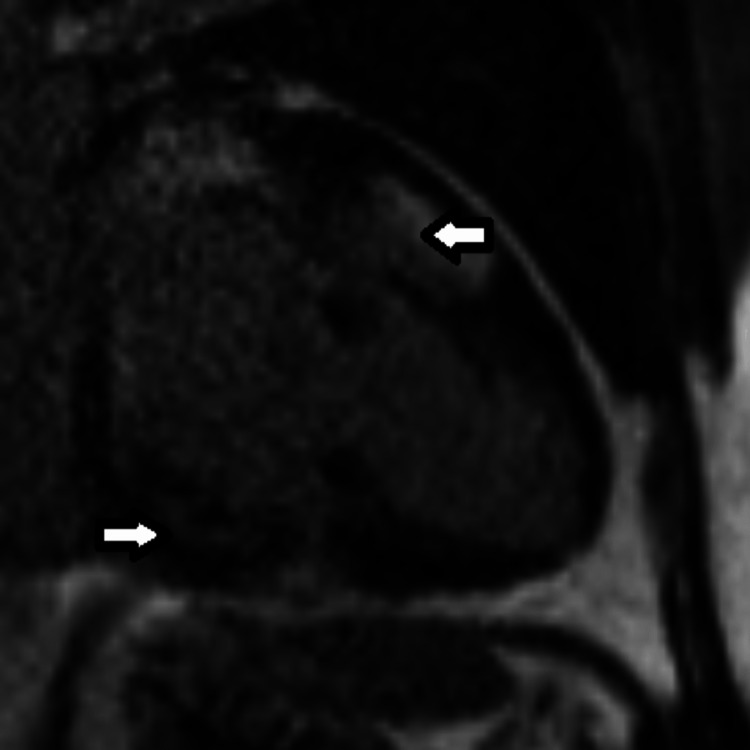
Cardiac MRI (Two-chamber view). Extensive areas of pathologic late gadolinium enhancement with mid myocardial and epicardial pattern with near complete transmural involvement, consistent with infiltrative heart disease (arrows).

**Figure 7:**
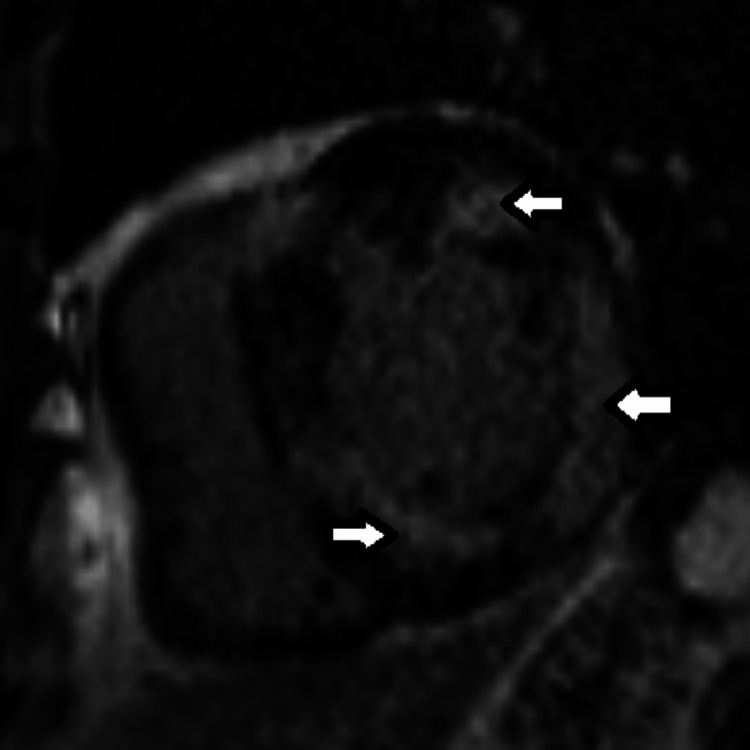
Cardiac MRI (Short axis). Multiple areas of pathologic late gadolinium enhancement with mid myocardial and epicardial pattern with near complete transmural involvement (arrows).

Cardiac magnetic resonance imaging (MRI) confirmed the presence of mild LV dilation [end diastolic volume (EDV) index 102 ml/m^2^, end-diastolic dimension (EDD) 6.5 cm] and moderate LV dysfunction (EF 38%) (Video 2). Tissue characterization by MRI showed extensive pathologic delayed gadolinium enhancement with patchy involvement of almost all myocardial segments (Figures 6, 7) with associated elevation in T1 times. These findings indicated diffuse myocardial fibrosis and were consistent with infiltrative heart disease.

**Video 2:** Cardiac MRI. Four chamber SSFP (steady-state free precession). Mild left ventricular dilation with severe systolic dysfunction. All visualized left ventricular segments appear depressed. Normal right ventricular size and systolic function.

**Figure 8:**
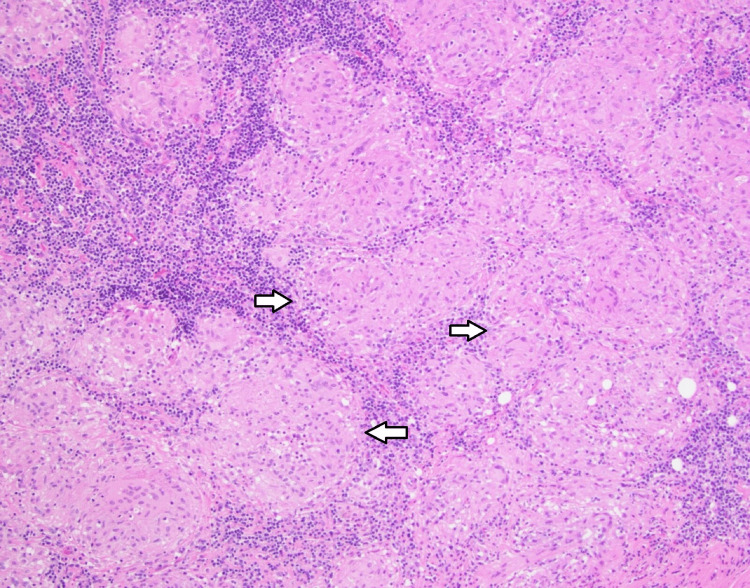
Lymph node biopsy (H&E, 10x magnification). Confluent, non-necrotizing, epithelioid granulomas (arrow).

**Figure 9:**
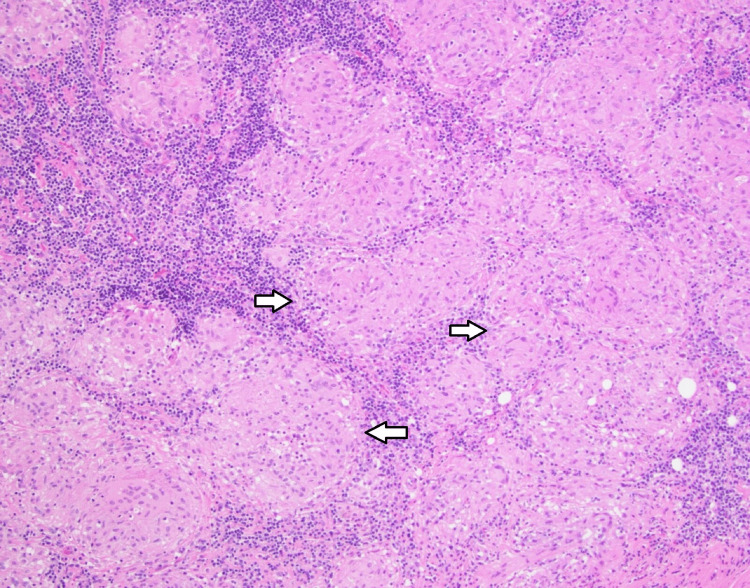
Lymph node biopsy (H&E, 20x magnification). Confluent, non-necrotizing, epithelioid granulomas (arrows).

**Figure 10:**
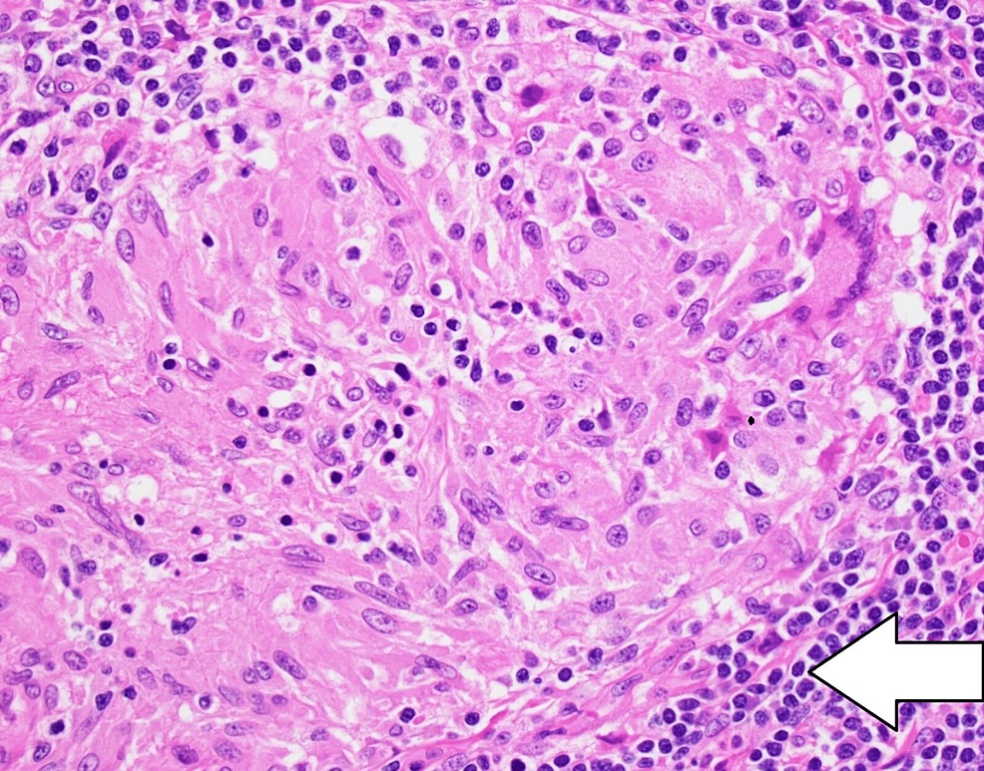
Lymph node biopsy (H&E, 40x magnification). Large granuloma (arrow).

A left axillary lymph node biopsy was performed and a pathologic examination showed granulomatous lymphadenitis without evidence of lymphoma (Figures 8-10).

The combination of the patient’s clinical history, findings on chest CT scan, whole body PET scan, echocardiogram, cardiac MRI, cardiac PET, bronchoalveolar lavage cultures, and lymph node biopsy was highly suggestive of sarcoidosis with cardiac involvement (cardiac sarcoidosis). Even though the infection with Mycobacterium avium complex was never confirmed, he completed a one-year course of antibiotics.

**Figure 11:**
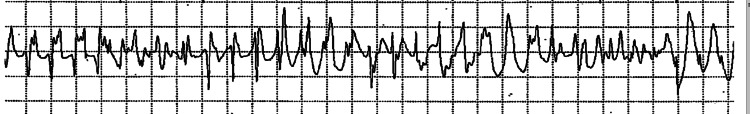
Automatic implantable cardioverter defibrillator (AICD) device interrogation. Ventricular tachycardia.

Ultimately, 18 months after the initial presentation, our patient was admitted to the hospital for Automatic Implantable Cardioverter Defibrillator (AICD) placement due to low ejection fraction three months after diagnosis of heart failure. During his hospitalization, almost at the time of discharge, he developed an episode of ventricular fibrillation/tachycardia for which his AICD shocked him (Figure 11). Based on his cardiac PET scan and MRI findings suggestive of active cardiac sarcoidosis, he was started on immunosuppression with pulse doses of steroids and infliximab. For medical treatment of systolic and diastolic heart failure, he was prescribed sacubitril/valsartan, metoprolol, and furosemide. He was discharged to home with pulmonary and cardiology follow-up appointments.

## Discussion

Sarcoidosis is a systemic disease characterized by the formation of non-necrotizing granulomas, primarily involving the lungs (prevalence 90%) and other organs such as the heart (prevalence 5-10%) [1-3]. The prevalence of sarcoidosis has been reported by Sikjaer et al. to be 77 per 100,000 persons and an incidence of 14.5 per 100,000 persons per year, with the initial presentation for both men and women between 30 and 39 years of age [4].

The clinical presentation of sarcoidosis is highly variable and depends on the organs involved. In the absence of significant organ involvement, sarcoidosis is often asymptomatic. Classic syndromes of presentation include Lofgren syndrome, lupus pernio, and erythema nodosum. Other manifestations include uveitis, optic neuritis, bilateral hilar adenopathy, pulmonary infiltrates, osteolysis, parotid involvement, hypercalcemia, hypercalciuria, hepatosplenomegaly, increased inflammatory activity in the heart (seen on MRI, PET, and gallium (Ga) scan) and new onset first degree AV block, among others.

The diagnosis of sarcoidosis is based on three major criteria, as described in the current sarcoidosis guidelines by the American Thoracic Society (ATS): a compatible clinical presentation, the presence of non-necrotizing granulomatous inflammation in a tissue sample (not always required), and exclusion of alternative causes of granulomatous disease [1]. Diagnostic evaluation for sarcoidosis includes pulmonary function tests, high-resolution CT of the chest (HRCT), bronchoscopy with bronchoalveolar lavage, and endobronchial ultrasound (EBUS).

Once the diagnosis of sarcoidosis is made, current sarcoidosis guidelines recommend baseline screening testing for organ involvement including slit-lamp eye exam, serum creatinine, serum alkaline phosphatase, serum calcium vitamin D levels, complete blood count, and ECG [4].

The clinical presentation of cardiac sarcoidosis includes palpitations, presyncope and syncope, and less frequently chest pain. Cardiac sarcoidosis should be suspected when the dyspnea appears to be disproportionate to the severity of apparent lung involvement. If clinical suspicion for cardiac sarcoidosis is high, transthoracic echocardiogram and cardiac MRI are recommended. If cardiac MRI is not available, or the cardiac MRI results are inconclusive, then PET CT scan is recommended [2, 5]. PET CT is particularly helpful when myocardial tissue characterization by MRI is difficult due to artifact from a previously implanted permanent pacemaker or AICD, or when renal dysfunction precludes the use of Gadolinium contrast agents [2, 5].

Typical ECG abnormalities include frequent ventricular ectopy, left or right bundle branch block, pathologic Q waves, left or right axis deviation, and ventricular arrhythmias.

Echocardiogram findings of cardiac sarcoidosis include left ventricular systolic or diastolic dysfunction. The presence of pulmonary hypertension and RV dysfunction can be seen in the presence of cardiac sarcoid but also with isolated lung involvement. If pulmonary hypertension is suspected, further characterization with right heart catheterization can also be obtained.

MRI findings of cardiac sarcoidosis include LV systolic dysfunction and evidence of infiltrative heart disease on myocardial tissue characterization. The presence of pathologic delayed Gadolinium enhancement with non-coronary patchy pattern, especially in the presence of associated elevated T1 times on corresponding T1 maps, is typical of cardiac sarcoidosis and has a high sensitivity (92-100%) and specificity (78-100%) for cardiac sarcoid [6-8].

Cardiac PET CT to evaluate for suspected cardiac sarcoidosis is usually performed with FDG-PET to detect inflammation along with a resting myocardial perfusion scan to exclude coronary artery disease. A mismatch pattern of perfusion defects and myocardial inflammation is characteristic of cardiac sarcoidosis and can be used to define different stages of the disease process [9-12].

The diagnosis of cardiac sarcoidosis can be difficult. The last set of diagnostic guidelines for cardiac sarcoidosis was published in 2019 by the Japanese Circulation Society (JCS) [5]. They propose the following diagnostic criteria:

1. Histological diagnosis group with positive myocardial biopsy consistent with non-caseating epithelioid granulomas.

2. Clinical diagnosis group after negative myocardial biopsy or decision to not pursue myocardial biopsy.

a. Epithelioid granulomas present in organs other than the heart and clinical findings suggestive of cardiac sarcoidosis.

b. Clinical findings suggestive of pulmonary or ophthalmic sarcoidosis along with clinical findings suggestive of cardiac sarcoidosis.

The criteria for cardiac involvement per the JCS are described in Table 1.

**Table 1:** Criteria for cardiac sarcoid involvement per the Japanese Circulation Society

Major criteria
High-grade atrioventricular block or fatal ventricular arrhythmia
Basal thinning of the ventricular septum or abnormal ventricular anatomy
Left ventricular contractile dysfunction or focal ventricular wall asynergy
Ga citrate scintigraphy or F-FDG PET (fluorodeoxyglucose-Positron Emission Tomography) reveals abnormally high tracer accumulation in the heart
Gadolinium-enhanced Magnetic Resonance Imaging reveals delayed contrast enhancement in the myocardium
Minor criteria
Abnormal ECG findings: ventricular arrhythmias, bundle branch block, axis deviation, or abnormal Q waves
Perfusion defects on myocardial perfusion scintigraphy
Endomyocardial biopsy: monocyte infiltration and moderate or severe myocardial interstitial fibrosis

In terms of treatment, corticosteroid therapy should be considered for patients with cardiac sarcoidosis who have a high-grade atrioventricular block, ventricular arrhythmias, or cardiac dysfunction. Second-line immunosuppressive therapies include cyclophosphamide, cyclosporine, azathioprine, methotrexate, thalidomide, hydroxychloroquine, pentoxifylline, and mycophenolic acid. Infliximab may be used for patients who do not respond to corticosteroids and at least one second-line therapy. Patients should also receive appropriate treatment for high-grade atrioventricular block, severe ventricular arrhythmia, and heart failure.

## Conclusions

Sarcoidosis is a systemic disease characterized by the formation of non-necrotizing granulomas, primarily involving the lungs (prevalence 90%) and other organs such as the heart (prevalence 5-10%). Once the diagnosis of sarcoidosis is made, current sarcoidosis guidelines recommend baseline screening tests for organ involvement including eye exam, serum creatinine, serum alkaline phosphatase and serum calcium vitamin D levels, complete blood count, and ECG. The presence of ECG abnormalities, particularly ventricular arrhythmias and conduction disease should prompt further cardiac evaluation including cardiology consultation, echocardiogram, F-FDG PET scan, and cardiac MRI. In cardiac sarcoidosis, F-FDG PET scan reveals abnormally high tracer accumulation in the heart. Gadolinium-enhanced MRI demonstrates delayed contrast enhancement in the myocardium in a non-coronary pattern, with associated elevation in T1 times, consistent with fibrosis.

In general, immunosuppressive therapy is effective in the treatment of cardiac sarcoidosis. Patients should also receive appropriate treatment for high-grade atrioventricular block (pacemaker), severe ventricular arrhythmia (AICD), and heart failure.
